# Heteronymous feedback from quadriceps onto soleus in stroke survivors

**DOI:** 10.21203/rs.3.rs-4540327/v1

**Published:** 2024-06-27

**Authors:** Cristian Cuadra, Steven L. Wolf, Mark A. Lyle

**Affiliations:** University at Buffalo; Emory University; Emory University

**Keywords:** Stroke, heteronymous reflexes, proprioceptive feedback, muscle spindle, recurrent inhibition, Golgi tendon organ, femoral nerve, quadriceps, abnormal coactivation

## Abstract

**Background:**

Recent findings suggest increased excitatory heteronymous feedback from quadriceps onto soleus may contribute to abnormal coactivation of knee and ankle extensors after stroke. However, there is lack of consensus on whether persons post-stroke exhibit altered heteronymous reflexes and, when present, the origin of increased excitation (i.e. increased excitation alone and/or decreased inhibition). This study examined heteronymous excitation and inhibition from quadriceps onto soleus in paretic, nonparetic, and age-matched control limbs to determine whether increased excitation was due to excitatory and/or reduced inhibitory reflex circuits. A secondary purpose was to examine whether heteronymous reflex magnitudes were related to clinical measures of lower limb recovery, walking-speed, and dynamic balance.

**Methods:**

Heteronymous excitation and inhibition from quadriceps onto soleus were examined in fourteen persons post-stroke and fourteen age-matched unimpaired participants. Heteronymous feedback was elicited by femoral nerve and quadriceps muscle stimulation in separate trials while participants tonically activated soleus at 20% max. Fugl-Myer assessment of lower extremity, 10-meter walk test, and Mini-BESTest were assessed in stroke survivors.

**Results:**

Heteronymous excitation and inhibition onsets, durations, and magnitudes were not different between paretic, nonparetic or age-matched unimpaired limbs. Quadriceps stimulation elicited excitation that was half the magnitude of femoral nerve stimulation. Femoral nerve elicited paretic limb heteronymous excitation was positively correlated with walking speed but did not reach significance because only a subset of paretic limbs exhibited excitation (n = 8, Spearman r = 0.69, P = 0.058).

**Conclusions:**

Heteronymous feedback from quadriceps onto soleus assessed in a seated posture was not impaired in persons post-stroke. Despite being unable to identify whether reduced inhibition contributes to abnormal excitation reported in prior studies, our results indicate quadriceps stimulation may allow a better estimate of heteronymous inhibition in those that exhibit exaggerated excitation. Heteronymous excitation magnitude in the paretic limb was positively correlated with self-selected walking speed suggesting paretic limb excitation at the higher end of a normal range may facilitate walking ability after stroke. Future studies are needed to identify whether heteronymous feedback from Q onto SOL is altered after stroke in upright postures and during motor tasks as a necessary next step to identify mechanisms underlying motor impairment.

## Introduction

Abnormal coactivation of lower limb muscles (i.e., synergies) is a common impairment after stroke, resulting in reduced walking and balance ability. For example, stroke survivors often exhibit abnormal coactivation of quadriceps and soleus during early-mid stance of walking that is associated with slower self-selected walking speed (1–3). Despite being a common problem related to poorer walking recovery, sensorimotor mechanisms that may contribute to the early activation of soleus during the stance phase of walking (1–5) in persons with stroke remain unknown.

Recent findings suggest altered heteronymous reflex pathways may contribute to abnormal Q and SOL coactivation (4–7). Heteronymous reflexes can contribute to lower limb muscle coactivation because excitatory length and inhibitory force-dependent feedback from Q can increase and decrease SOL motor output (8–12). For example, stimulating the femoral nerve normally causes a brief excitation of SOL attributed to Ia afferent feedback that is immediately followed by a longer duration inhibition (5, 8, 9, 11, 12). Thus, abnormal coactivation of Q and SOL could result from too much heteronymous excitation and/or too little inhibition due to impaired descending control after stroke. Indeed, a few studies have reported a marked increase in heteronymous excitatory magnitude and duration from Q onto SOL with reduced or absent inhibition in the paretic limb of stroke survivors (4, 5). Even though the heteronymous feedback was evaluated while sitting, an increased heteronymous excitation was moderately correlated with abnormal coactivation of Q and SOL during walking (5), as well as poorer coordination as assessed by the lower extremity coordination test and motor recovery using the Chedoke-McMaster Stroke Assessment (4). Given that other studies have not demonstrated a significant increase in heteronymous excitation from quadriceps onto soleus after stroke, nor a relation between heteronymous feedback and clinical measures (13, 14), further examination is warranted to clarify these conflicting findings.

An additional knowledge gap is that the standard approach of eliciting heteronymous feedback with femoral nerve stimulation could not resolve whether the increased heteronymous excitation from Q onto SOL post-stroke observed by Dyer et al. 2009, 2014 was due to excessive Ia excitatory feedback and/or reduced inhibition. Femoral nerve stimulation elicits heteronymous excitation by activating Ia afferent axons with mono- and polysynaptic contacts onto SOL motoneurons, whereas heteronymous inhibition is attributed to antidromic propagation along motor axons acting on Renshaw cells (i.e., recurrent inhibition) and probably from Ib afferent or muscle twitch-elicited activation of Golgi tendon organ receptors acting on interneurons that inhibit SOL motoneurons (11, 12, 15, 16). Because Ia afferent axons have a lower stimulation threshold and faster conduction velocity compared to Golgi tendon organ and motor axons (17–20), a sensorimotor impairment of heteronymous inhibition is difficult to identify in the presence of a large heteronymous excitation which always precedes the inhibition. However, recent evidence suggests Q muscle stimulation is a viable alternative to nerve stimulation if the goal is to examine heteronymous inhibition without undue influence from heteronymous excitation (11, 12, 16, 21). Q muscle stimulation elicits heteronymous inhibition with the same onset and slightly reduced magnitude and duration compared to femoral nerve stimulation in adults without neurological injury (11, 12). A key advantage of eliciting heteronymous feedback with Q muscle stimulation is that heteronymous excitation of SOL is significantly reduced compared to femoral nerve stimulation, presumably due to reduced direct activation of Ia sensory axons (9, 11, 12). Thus, eliciting heteronymous feedback with Q muscle stimulation provides an opportunity to identify impaired heteronymous inhibition after stroke, if present.

Accordingly, the purposes of this study were to identify whether persons with stroke exhibit sensorimotor impairments in heteronymous excitation and inhibition from Q onto SOL, and whether heteronymous reflex magnitudes are related to clinical measures of lower limb recovery (Fugl-Myer), self-selected walking-speed, and dynamic balance (MiniBEST). To clarify whether impairments in heteronymous excitation and/or inhibition are present after stroke, femoral nerve and quadriceps muscle stimulation were used to elicit heteronymous feedback onto SOL motor ouput. Based on the work of Dyer et al. 2009, 2014, we hypothesized femoral nerve-elicited heteronymous excitation will be significantly greater and inhibition significantly reduced in persons with stroke compared to age-matched controls. We expected that, when compared to femoral nerve stimulation, Q muscle elicited heteronymous excitation would be decreased in persons with and without stroke. We further predicted Q muscle belly elicited heteronymous inhibition would be significantly decreased in stroke survivors compared to age-matched controls. Lastly, we expected a significant correlation between increased heteronymous excitation and poorer performance on clinical measures.

## Methods

Fourteen stroke survivors (9 males and 5 females, age between 40 to 76) and fourteen age-matched control participants (7 males and 8 females, age between 32 to 75 years) were recruited for this study. Participants provided written informed consent in accordance with procedures approved by the Emory University Institutional Review Board. Persons with stroke were eligible if they had a single stroke event at least 6 months prior to participation and no signs of neglect. All participants could sustain a voluntary isometric plantarflexion contraction for at least 5–10 seconds to complete the experimental task. Participants were excluded if they had a recent history of lower limb injury or neuromotor disorder other than stroke, inability to comprehend instructions, or implanted devices (pacemaker, CSF pump, metallic implants).

### Equipment

**Figure 1:**
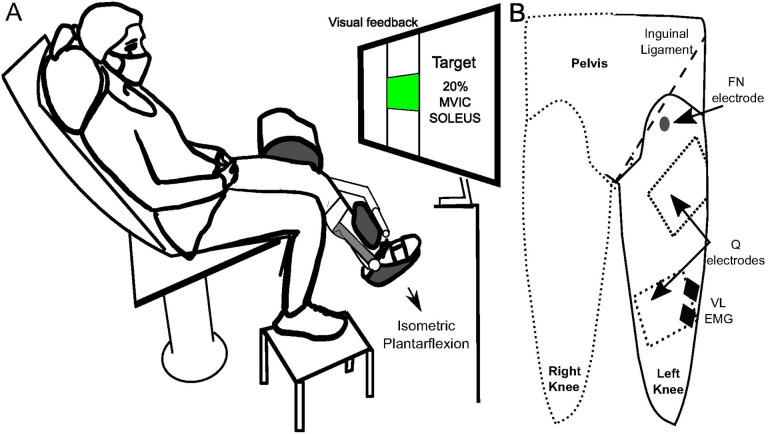
A) Schematic depiction of the experimental setup with participants’ leg securely fastened against the dynamometer arm. B) Anterior view illustration showing the placement of stimulation and recording electrodes on the left thigh (rectangular anode positioned on the posterior buttock is not shown). The dashed rectangles correspond to the positioning of Q stimulation electrodes. VL, vastus lateralis; FN, femoral nerve.

All recordings were obtained at a sampling rate of 2000 Hz using an MP150 acquisition unit (Biopac Systems Inc, Goleta, CA, USA). Surface electromyography (EMG) was recorded from the soleus (SOL) and vastus lateralis (VL) muscles utilizing Ag-AgCl electrodes (2.2 × 3.5 cm; Vermed, Buffalo, NY, USA). Prior to electrode placement, the skin was abraded with gauze and cleansed with isopropyl alcohol. The SOL electrodes were positioned along the midline of the posterior aspect of the shank just below the gastrocnemius muscles, and the VL electrodes were placed on the distal third of the muscle (Fig. 1). EMG were amplified (x500–1000) and hardware filtered (10–1000 Hz) (AMT-8, Bortec Biomedical Ltd, Canada). Nerve and muscle stimulation procedures were completed with STM100C stimulators (Biopac System Inc, CA, USA).

### Experimental Procedures

Persons with stroke attended separate testing sessions to examine heteronymous feedback from Q onto SOL for the paretic and contralateral leg, but all testing was completed in a single session for control participants. For stroke participants, the number of days between sessions were 7.64 ± 6.4 days. Subjects were seated on a dynamometer chair (Humac Norm, CSMI, Inc, Worcester, MA) with the knee flexed to 40° and the hip to 60°. The dynamometer axis of rotation was aligned with the knee joint, and the dynamometer pad was secured against the distal tibia with rigid straps. An ankle immobilizer (United Orthopedics ROM Walker, Fort Wayne, IN, USA) was applied to the lower leg to enable isometric contractions with the ankle in a neutral position.

### Maximal voluntary isometric contractions

To identify maximal SOL electromyography (EMG), the seated participants performed three maximal effort isometric plantarflexion contractions (MVIC) lasting 5 seconds. Verbal encouragement and real-time SOL EMG feedback were provided to optimize participant effort. The SOL EMG was bandpass filtered (10–500 Hz), rectified, and a 400 ms moving average used to identify the peak SOL value for each trial. The maximal SOL EMG was determined as the mean of the three trials and subsequently used to provide visual feedback during reflex testing.

### Assessment of heteronymous feedback from quadriceps onto soleus

Heteronymous feedback from quadriceps was elicited by separately stimulating the femoral nerve and quadriceps muscle belly. The femoral nerve (FN) was stimulated with the cathode placed in the area medial to the rectus femoris and approximately 1 cm distal to the inguinal ligament (1 ms pulse width, 2.2 × 3.5 cm). The cathode was moved in this area to identify the stimulation location producing a palpable contraction in VL, rectus femoris (RF), and vastus medialis (VM) with the lowest current. The anode was placed on the posterolateral buttock (7.5 × 13 cm, ValuTrode, Axelgaard Co., Ltd, CA, USA). Motor threshold was identified by increasing the stimulation intensity until an M-wave was visible from VL.

Quadriceps muscle belly stimulation was achieved with two rectangular self-adhesive electrodes (7.5 × 13 cm, ValuTrode, Axelgaard manufacturing Co., Ltd, CA, USA) placed on distal (VM) and proximal (VL-RF) motor point areas identified with a pen electrode (22). Stimulation comprised a doublet (two unipolar pulses, 50 μs pulse width, 5 ms interpulse interval) to leverage the catchlike property of muscle (23) and a short pulse width to minimize the activation of sensory axons (19, 24, 25). Quadriceps motor threshold was considered the lowest stimulation current that resulted in a palpable contraction in VL, rectus femoris (RF), and vastus medialis (VM) (26).

Heteronymous feedback from quadriceps onto soleus was first examined by stimulating the femoral nerve at 2x motor threshold (60.5 ± 16.4 mA) while participants targeted 20% SOL MVIC. Participants were provided visual feedback of the SOL EMG target displayed as 20 ± 5% via a vertical bar. Each person practiced until they were comfortable achieving the background SOL EMG target. Stimulation of the femoral nerve (or quadriceps muscle belly) was software triggered when participants maintained the 20 ± 5% SOL magnitude for at least 2 seconds. Thirty repetitions were recorded with a minimum rest period of 10 seconds between repetitions (27, 28). After completing the femoral nerve stimulation repetitions, heteronymous feedback onto soleus EMG was examined by stimulating the quadriceps muscle belly using the same procedures as described above for femoral nerve stimulation. The Q stimulation intensity was chosen to match the peak torque produced by FN stimulation (2.04 ±0.33x motor threshold; range 1.3–3 x motor threshold; 78±14.6 mA). Thirty repetitions were recorded with a minimum rest period of 10 seconds between repetitions.

#### Clinical evaluation:

Stroke participants underwent clinical assessments to evaluate lower limb function on a separate day from the reflex testing sessions. The Fugl-Meyer assessment (FMA) total motor score was used to assess motor impairment. The lower extremity section comprises a total possible score of 34 with higher numbers reflecting lesser motor impairment (29), Self-selected walking speed was assessed with the 10-m walking test and quantified as the average of three trials. Dynamic balance ability was assessed with the Mini Balance Evaluation Systems Test (MiniBESTEST) (30).

### Data Analysis

All data were analyzed offline using custom scripts written in MATLAB R2021a (MathWorks, Natick, MA). Heteronymous excitatory and inhibitory feedback from Q onto SOL resulting from femoral nerve (FN) and quadriceps (Q) muscle belly stimulation were assessed after bandpass filtering (10–500 Hz, zero phase shift) and rectification. The rectified SOL EMG was normalized to each participant’s maximum voluntary isometric contraction (MVIC) value and ensemble averaged across repetitions for both FN and Q stimulation. The background SOL EMG prior to stimulation was calculated as mean and standard deviation (SD) over a 400 ms period preceding stimulation.

The mean and SD of the background SOL EMG for each stimulation condition were used to identify excitation and inhibition onset, duration, and magnitudes as previously described (11, 12). Excitation onset was identified as the point in time when the SOL EMG activity exceeded 1 SD above the mean for ≥ 2 ms while the end of excitation was identified when the SOL EMG activity fell below the 1 SD line for ≥ 2 ms. Only excitatory responses with an onset ≥ 23 ms were considered as originating from heteronymous Ia facilitation (10, 31). Onset and termination of inhibition were determined as the SOL EMG moving 1 SD below the mean background SOL EMG for ≥ 2 ms and returning above the 1 SD line for ≥ 2 ms, respectively (11, 12, 32). The durations of excitation and inhibition were calculated as the difference between effect onset and termination. Excitation and inhibition magnitudes were determined as the area relative to background SOL EMG using trapezoidal numerical integration (trapz in Matlab). The peak knee extensor torque in response to FN and Q stimulation were extracted from time–torque traces.

### Statistical analysis

Statistical analyses were conducted using R Statistical Software (© 2009–2020 RStudio, version 1.3.1073, PBC). Descriptive statistics presented in the text and figures are shown as mean ± standard deviation. The normality assumption was inspected with quantile-quartile plots for each variable and condition and evaluated with Shapiro-Wilks test. Repeated-measures ANOVAs were run using the MIXED procedure with participant treated as a random factor. The F-values were computed using the Kenward–Roger method and compound symmetry variance–covariance structure to test the hypotheses at p<0.05. Pairwise contrasts were evaluated with Bonferroni correction.

To evaluate the heteronymous feedback from Q onto SOL EMG, separate two-way repeated-measures ANOVAs were used to examine the effect of stimulation location (i.e., FN and Q muscle stimulation) and limb(i.e., paretic, non-paretic, and control) on heteronymous excitation and inhibition magnitude, onset, and duration. Additional statistical tests were used to evaluate experimental control of participants’ stimulation evoked torque and whether the 20% SOL background EMG was consistently achieved. Separate two-way repeated measures ANOVAs were used to identify whether stimulation evoked torque and background SOL EMG were similar across stimulation locations (FN and Q muscle belly stimulation) and limbs (paretic, nonparetic, and control limbs).

Lastly, Spearman’s rank correlation coefficients were used as a conservative estimate of the associations between clinical measures (FMA total motor scores, self-selected walking speed, and MiniBEST), and the associations between clinical measures and heteronymous excitation and inhibition of the paretic limb separately for FN and Q stimulation.

## Results

### Heteronymous excitation

**Figure 2:**
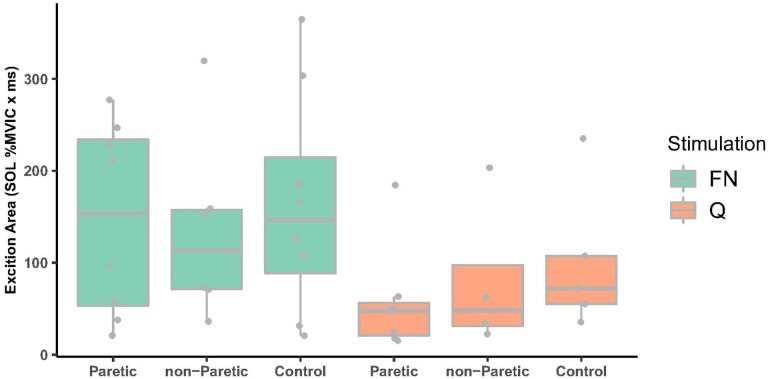
Heteronymous excitation area comparing paretic, non-paretic and control limbs for FN (green boxplots) and Q stimulation (orange boxplots). FN stimulation resulted in larger excitation compared to Q stimulation (*P-value*=0.018). FN, femoral nerve, Q, quadriceps

The frequency of participants exhibiting excitation was similar across paretic, nonparetic, and control limbs for femoral nerve (n= 8, 6, 8) and quadriceps muscle belly stimulation (n= 7, 4, 5). For those exhibiting excitation, femoral nerve stimulation elicited larger excitation onto SOL compared to Q muscle belly stimulation (*F*_1,20.11_= 6.61, *P-value*= 0.018, 149.90 ±105.64 vs. 76.90 ± 69.55 % MVIC ‘ ms, Figure 2). However, excitation magnitude was not different between paretic, non-paretic, and control limbs (*F*_2,22.60_=0.27, *P-value*= 0.769, 105.44 ± 95.27, 113.47 ± 94.76, and 139.38 ±107.99 % MVIC ‘ ms, respectively), nor was excitation onset (*F*_22334_=0.13, *P-value*= 0.880, 31.7± 5.71 ms, 32.3± 2.28 ms, and 31.4 ± 4.55 ms, respectively) or duration (F_2,24.6_= 0.13, *P-value*=0.876, 6.73 ± 4.61, 7.86 ± 4.45, and 6.85 ± 3.26 ms, respectively).

### Heteronymous inhibition

**Figure 3:**
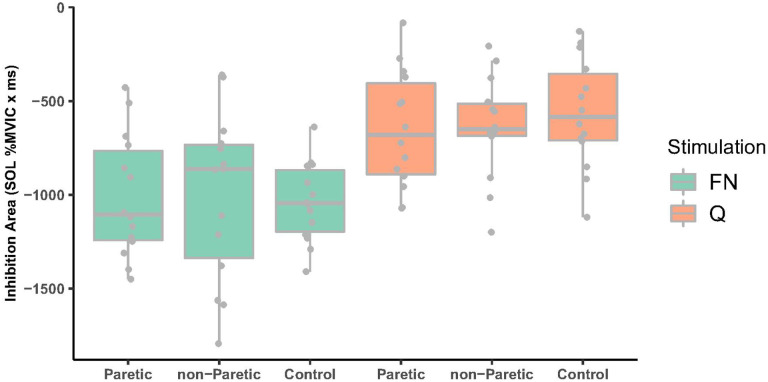
Heteronymous inhibition area comparing paretic, non-paretic and control limbs for FN (green boxplots) and Q stimulation (orange boxplots). FN nerve stimulation resulted in larger inhibition compared to Q (*P-value*<0.001) but there were no differences in inhibition between paretic, non-paretic, and control limbs (P =0.96). FN, femoral nerve, Q, quadriceps.

FN stimulation elicited larger heteronymous inhibition of SOL compared to Q muscle belly stimulation (Figure 3, *F*_1,52.46_=52.92, *P-value*<0.001; −1018.01 ± 334.07 and −617.83 ± 286.65 %MVIC ‘ ms, respectively), primarily due to FN stimulation eliciting a longer inhibitory duration (*F*_1,52.75_= 30.79, *P-value*<0.001, 82.32 ± 24.37 ms, and 56.73 ± 24.59 ms, respectively). However, heteronymous inhibition magnitudes were not different between paretic, nonparetic and control limbs (*F*_2,46.95_ = 0.04, *P-value*= 0.961, −829.94 ± 362.26, −821.77 ± 408.30, and −802.04 ± 347.04 % MVIC ‘ ms, respectively), nor was the inhibitory duration (*F*_2,47.9_=0.45, *P-value*=0.640, overall mean 69.5 ± 27.5 ms). The onset of heteronymous inhibition was significantly earlier for FN compared to Q stimulation, but the difference was only 3 ms (*F*_1,52.17_=8.51, *P-value*=0.005, overall mean 39.95 ± 4.96 and 42.16 ± 5.24 ms, respectively).

### Clinical assessments and relation with heteronymous reflexes.

The correlations among the FMA total motor scores, self-selected walking speed, and MiniBEST were weak to moderate and not significant (n=14, MiniBEST vs walking speed, Spearman r = 0.51, P=0.062; MiniBEST vs FMA total motor scores , Spearman r = 0.46, P=0.1; FMA total motor scores vs walking speed, Spearman r = 0.29; P=0.32). Although not significant due to only a subset of participants exhibiting excitation, heteronymous excitation in the paretic limb was moderately correlated with self-selected walking speed (n=8; Spearman r = 0.69, P=0.058). The paretic limb heteronymous excitation and inhibition elicited by FN or Q stimulation were weak to moderately correlated with FMA total motor scores or MiniBEST tests and not significant (Spearman r = 0.02 – 0.5, P = 0.07–0.96)

### Background SOL EMG and torque were consistent across conditions.

The background SOL EMG prior to FN or Q stimulation approximated the SOL MVIC target goal with an overall mean of 19.2 ± 1.93 % SOL MVIC. SOL background EMG did not differ between paretic, nonparetic, and control limbs (*F*_2,47.48_ = 0.40, *P-value*= 0.675) or between FN and Q stimulation (*F*_1,52.62_ = 0.55, *P-value*=0.582). Similarly, stimulation evoked torque magnitudes were consistent across paretic, nonparetic, and control limbs (F_2,4.97_ = 0.15, *P-value*= 0.865) and stimulation locations (*F*_1,52.14_ = 0.08, *P-value*= 0.776).

## Discussion

Lower limb heteronymous reflexes can influence motor coordination because excitatory and inhibitory feedback from a muscle can increase and decrease motor output of other muscles (8–12, 15, 17, 18, 25, 31, 33, 34). Therefore, abnormal lower limb motor coordination such as Q and SOL coactivation frequently observed after stroke during walking could arise, in part, from alterations to the strength of excitatory and inhibitory heteronymous feedback (4–7, 13). This study examined whether persons with stroke exhibit sensorimotor impairments in heteronymous excitation and inhibition from Q onto SOL, and whether heteronymous reflex magnitudes are related to lower limb motor impairment, walking speed, and dynamic balance. In contrast to our hypotheses, the magnitudes of heteronymous excitation and inhibition from Q onto SOL in stroke survivors’ paretic limbs were not different when compared to the nonparetic or age-matched unimpaired limbs. Likewise, no significant correlations were observed between heteronymous excitation or inhibition magnitudes and clinical measures. However, greater heteronymous excitation in the paretic limb was nonetheless moderately correlated with self-selected walking speed suggesting the possibility that persons with stroke exhibiting heteronymous excitation on the higher end of normal walk faster. Collectively, these findings suggest heteronymous feedback from Q onto SOL in the stroke survivors was not abnormal as assessed in a seated posture in this study.

### Heteronymous excitation from Q onto SOL was not impaired in persons with stroke

To date, only 3 research groups had previously examined heteronymous feedback from Q onto SOL in persons with stroke (4–7, 13, 14). The primary focus has been on the magnitude of heteronymous excitation because excessive excitation could contribute to the abnormal Q and SOL coactivation frequently observed in stroke survivors during walking (3). In support of this premise, one research group observed abnormally increased heteronymous excitation in persons with stroke from Q onto the SOL H-reflex (4) and ongoing tonic SOL EMG (5, 7). In contrast, the present study found that femoral nerve elicited heteronymous excitation measured as change in tonic SOL EMG was not greater in the paretic compared to the nonparetic or age-matched control limbs. Similarly, Faist et al. found that heteronymous excitation of the SOL H-reflex did not differ between paretic and control limbs. Lastly, Takahashi et al. 2022 reported a modest heteronymous facilitation of less than a 20% increase from Q onto the SOL H-reflex in stroke survivors which is comparable to the facilitation found in a sample of young adults without stroke (11). Despite no differences in heteronymous excitation between paretic, nonparetic and control limbs in the present study, the femoral nerve elicited excitation in the paretic limb was moderately correlated (Spearman r= 0.69, P=0.058) with walking speed suggesting that heteronymous excitation, at least at the top end of a “normal” range, may facilitate walking ability in stroke survivors. In contrast, Dyer et al. 2014 found that abnormally increased heteronymous excitation was correlated with earlier coactivation of the SOL and vastus lateralis muscles during walking in their sample of stroke survivors (r=0.73), but the relation to walking speed was not reported (5). Further work is needed to clarify whether heteronymous excitation magnitudes facilitate or impede walking ability after stroke. An important finding in this study was that heteronymous excitation magnitudes in paretic, nonparetic, and control limbs elicited by Q muscle belly stimulation were about half of that elicited by femoral nerve stimulation (see figure 2). This finding adds to the growing evidence that Q muscle belly stimulation elicits reduced heteronymous Ia excitatory feedback compared to femoral nerve stimulation. Q muscle belly stimulation was used in this study to better estimate whether heteronymous inhibition is impaired in the presence of abnormally large excitation. While heteronymous excitation was not abnormally increased in stroke survivors in this study, the study findings suggest nonetheless that Q muscle belly stimulation could be helpful to quantify heteronymous inhibition in persons that exhibit markedly increased excitation.

### Heteronymous inhibition from Q onto SOL was not impaired in persons with stroke

Despite most studies to date focusing on heteronymous excitation, a sensorimotor impairment of heteronymous inhibition could also contribute to abnormal motor coordination after stroke. Several studies by the same research group found reduced heteronymous inhibition in paretic compared to nonparetic and control limbs (4, 5). In contrast to our hypothesis and Dyer et al. (4–6), the present study found no differences in heteronymous inhibition magnitude or duration between stroke or age-matched control limbs, whether elicited by femoral nerve or muscle belly stimulation. In addition, the magnitudes of heteronymous inhibition were weakly correlated with clinical measures, consistent with the findings of Dyer et al 2009, 2014. The findings from the present study indicate no sensorimotor impairment in heteronymous inhibition from Q onto SOL in the persons with stroke as assessed in the seated posture in this study.

### Comparison of heteronymous excitation and inhibition with prior work in younger adults

Several novel insights about heteronymous feedback are suggested by comparing the findings of this study with prior work using the same methods in younger adults. The magnitude and duration of femoral nerve elicited heteronymous excitation onto SOL EMG found in this sample of older adults with and without stroke (149.90 ±105.64 %MVIC ‘ ms, 7–8 ms) was greater than that found in adults with mean age of 26 (79.96 +/− 61.8 %MVIC ‘ ms, 4–6 ms duration) (12). Interestingly, the femoral nerve elicited inhibitory magnitude was also larger in the older adults in this study compared to the younger adults (−1018.01 ± 334.07 vs −773 ± 277 %MVIC ‘ ms). The larger excitation and inhibition magnitudes observed in the older cohort of this study compared to younger adults was surprising, because heteronymous excitation (35) and reciprocal inhibition (i.e., a heteronymous circuit) have been shown to steadily decrease from 20 to 80 years old (36); however, the decreased magnitudes of excitation and inhibition were quantified as the change in the SOL H-reflex size and therefore offer a potentially different perspective compared to change in ongoing SOL EMG. Given that the change in ongoing SOL EMG reflects the net change in recruitment of an already active SOL motoneuron pool, and that the normal aging process involves an expanding of motor unit innervation number due to denervation and reinnervation of muscle fibers (37, 38), we speculate that the larger heteronymous excitation and inhibition in older adults could reflect recruiting and de-recruiting SOL motor units with a higher innervation ratio compared to young adults. Further study in a larger sample with motor unit analyses would be needed to test this hypothesis in the future.

### Methodological Considerations

To date, all studies have examined heteronymous feedback between Q and SOL in persons with stroke while in a seated posture using similar methods. Thus, subtle methodological choices or patient characteristics are likely responsible for only a subset of studies observing abnormal heteronymous excitation and inhibition in stroke survivors. One methodological difference between studies was the tonic SOL EMG magnitude participants were required to achieve when assessing heteronymous feedback. In Dyer et al. 2014, participants held a tonic 30% SOL EMG during heteronymous reflex testing, whereas in the present study participants targeted 20% SOL EMG. Perhaps the greater SOL contraction effort heightened overall excitability and contributed to the increased excitatory responses observed by Dyer et al. 2014; however, conflicting results were also found among studies when examining heteronymous excitation onto SOL H-reflex while SOL was inactive(4, 13, 14). Patient characteristics such as age, chronicity, and severity could contribute to differences between studies, but no obvious difference is apparent. While stroke survivors were younger in Dyer et al. 2009, 2014 (mean age around 50 years) compared to the present study (59 years on average), other studies that did not find increased heteronymous excitation enrolled stroke survivors with mean age of 50 (13) and 63 years (14). Stroke chronicity and severity seem to be reasonably similar across studies though difficult to formally assess due to different clinical measures and heterogeneity. Brain lesion size and location is an additional factor that has recently proven to account for heterogeneity in motor function after stroke and response to intervention (39–41). Future work would therefore benefit from considering the influence of brain lesion load on heteronymous reflex magnitudes and motor coordination.

The inconsistent heteronymous reflex findings in persons with stroke as assessed when sitting warrant future examination during other postures and tasks. Reflex magnitudes are task and phase dependently modulated to facilitate task goals (33, 42–53) For example, soleus H-reflex magnitude is normally decreased in standing compared to supine, and the H-reflex magnitude increases during mid-late stance phase of walking and decreases during the swing phase. In neurotypical adults, heteronymous excitation from Q onto SOL decreases in standing and walking compared to sitting (33, 48). In addition, heteronymous inhibition from Q onto SOL decreases when performing a task that requires both Q and SOL to work synergistically to maintain the posture such as when squatting (33). Heteronymous excitation from the tibialis anterior onto the Q has been shown to be increased in persons with stroke during walking (54). Thus, examining heteronymous feedback from Q onto SOL in persons with stroke during upright postures and walking is expected to more directly clarify whether altered reflexes contribute to motor impairments.

## Conclusions

In summary, this study contributes to the growing body of knowledge examining heteronymous reflexes and motor impairment after stroke. In this study, we found heteronymous excitation and inhibition from Q onto SOL was not different between paretic, nonparetic and age-matched control limbs in a seated posture. Nonetheless, the magnitude of heteronymous excitation in the paretic limb was positively correlated, approaching statistical significance (p = .058), with self-selected walking speed suggesting excitation in the higher range of normal may facilitate walking ability after stroke. We propose future studies are needed to identify patient characteristics that contribute to altered heteronymous reflexes and to determine whether heteronymous feedback from Q onto SOL is more consistently altered after stroke in upright postures and during motor tasks as a necessary next step to identify mechanisms of motor impairment.

## Figures and Tables

**Table 1:** Demographic and clinical data for all stroke participants

Participant	Age	Gender	Paretic side	Year since stroke	FMA Total Motor Score (max 34)	Self-selected Gait Speed (m/s)	MiniBest (max 28)
S01	58	M	R	3	28	1.3	24
S02	59	M	L	3	27	1.2	17
S03	45	M	L	3	23	0.6	15
S04	68	F	R	3	29	0.6	22
S05	40	F	L	4	29	0.4	11
S06	51	M	R	4	31	0.8	21
S07	60	M	L	4	32	1.0	27
S08	55	M	L	4	29	1.0	26
S09	69	F	L	4	30	1.0	13
S10	57	F	L	4	23	0.7	19
S11	76	M	L	4	33	1.1	26
S12	73	M	L	4	26	0.6	10
S13	55	F	R	4	30	0.4	18
S14	65	M	L	4	24	0.5	19


FMA: Fugl-Meyer Assessment.

## Abbreviations

FN: femoral nerve
Q: quadriceps
SOL: soleus
CSF: cerebrospinal fluid
FMA: Fugl-Meyer assessment
EMG: electromyography
VL: vastus lateralis
MVlC: maximum voluntary isometric contraction
RF: rectus femoris
VM: vastus medialis
SD: standard deviation
